# Supplementary material for: A high-resolution mRNA expression time course of embryonic development in zebrafish
Source: eLife. 2017 Nov 16;6:e30860. doi: 10.7554/eLife.30860 (PMC5690287; doi:10.7554/eLife.30860)
Supplement: Supplementary file 6. [file elife-30860-supp6.zip › biolayout-clusters-files/Cluster109.html]

Cluster109


# Cluster109: Detail

### Go to ZFA detail

## GO

| GO ID | Description | Domain | Annotated | Expected | Observed | Adjusted p-value | Genes | Ensembl IDs |
| --- | --- | --- | --- | --- | --- | --- | --- | --- |
| GO:0006364 | rRNA processing | biological\_process | 85 | 0.09 | 4 | 0.00028 | ncl utp15 wbp11 exosc4 | ENSDARG00000002710 ENSDARG00000002720 ENSDARG00000101411 ENSDARG00000103432 |
